# Rheumatic Diseases in China

**DOI:** 10.1186/ar2368

**Published:** 2008-01-31

**Authors:** Qing Yu Zeng, Ren Chen, John Darmawan, Zheng Yu Xiao, Su Biao Chen, Richard Wigley, Shun Le Chen, Nai Zheng Zhang

**Affiliations:** 1 Department of Rheumatology, the 1st Affiliated Hospital, Shantou University Medical College, 22 Xinling Road, Shantou, 515041 Guangdong, China; 2 Chenghai Municipal Hospital, Huancheng Bei Road, Chenghai District, Shantou, 515800 Guangdong, China; 3 World Health Organization Collaborating Center, Community-based Epidemiology, Treatment, and Prevention of Rheumatic Disease, Indonesian Rheumatic Center, 7 Jalan Seroja Dalam, Jakarta-Semarang, 50136 Indonesia; 4 World Health Organization Collaborating Center, Epidemiology of Rheumatic Disease, Research Laboratory, Palmerston North Hospital, 240 Park Road, Palmerston North, New Zealand; 5 Department of Rheumatology, Renji Hospital, Medical Science of Shanghai Jiaotong University, 145 Shandong Middle Road, Shanghai, 200001 China; 6 Department of Rheumatology, Peking Union Medical College Hospital, 1 Shuaifuyuan, Beijing, 100730 China

## Abstract

**Introduction:**

Epidemiological studies of rheumatic diseases have been conducted during the past 20 years in China. The aim of this study was to clarify prevalence rates of common rheumatic diseases in China.

**Methods:**

Relevant reports of population-based surveys conducted from 1980 to 2006 were retrieved. Studies using the World Health Organization-International League of Associations for Rheumatology COPCORD (Community Oriented Program for Control of Rheumatic Diseases) protocol and those that did not employ this protocol but were published in recognized journals were identified and analyzed.

**Results:**

Thirty-eight surveys including 241,169 adults from 25 provinces/cities were pooled for analysis. The prevalence of rheumatic complaints ranged from 11.6% to 46.4%, varying by locality, study protocol and age of the people surveyed. Prevalence of symptomatic osteoarthritis (OA) varied from 5.1% to 20.8%, with common sites of involvement being the lumbar spine, knee joint and cervical spine. Compared with rates of radiographic and symptomatic knee OA in the USA, elderly men in Beijing exhibited similar prevalence rates and elderly women exhibited a higher prevalence. The prevalence of hip OA and hand OA was much lower in Chinese than in Caucasian populations, but both kinds of OA were more common in coal miners. The prevalence of ankylosing spondylitis ranged from 0.2% to 0.54% among Han ethnic Chinese and were lower among mixed ethnic populations. The prevalence of psoriatic arthritis ranged from 0.01% to 0.1%, and that of reactive arthritis was 0.02%; undifferentiated spondyloarthropathy was identified in 0.64% to 1.2% of the individuals included in the surveys. The prevalence of rheumatoid arthritis (RA) ranged from 0.2% to 0.93%, with the highest rate being reported from a Taiwan urban area. In mainland China there were no significant differences in prevalence of RA between the northern and southern parts of China, or between different ethnic groups. The prevalence of hyperuricemia increased after the 1980s. The prevalence of gout was found to have increased in recent decades from 0.15% to 1.98%, apart from in the Taiwan aborigines, among whom the highest prevalence rate of 11.7% was recorded. The prevalence of primary Sjögren's syndrome in Beijing was 0.77% by the Copenhagen criteria and 0.33% by the San Diego criteria. The prevalence of soft tissue rheumatism was 2.5% to 5.7%. Fibromyalgia was seldom observed in China.

**Conclusion:**

Rheumatic diseases are common in China. The prevalence of rheumatic complaints varied with the locality surveyed. The prevalence of OA is comparable with that in Western countries but varies in terms of joint involvement. The prevalence of ankylosing spondylitis is similar to that in Caucasians. Except in Taiwan, the prevalence of RA in China is lower than that in developed countries. The prevalence of hyperuricemia and gout increased after the 1980s, but it remains lower than that in developed countries. More studies are required to evaluate prevalence rates among minority groups in the west and northwest parts of China, and further study is needed to address fibromyalgia in China.

## Introduction

Rheumatology is a relatively new subspecialty of medicine in China. The first rheumatology unit in China was established in 1980 in the Peking Union Medical College Hospital. Before that, little was known about the epidemiology of rheumatic diseases in China.

Initiated by International League of Associations for Rheumatology (ILAR) and its then president Professor EP Engleman, and the president of the Chinese Association of Rheumatology, Professor NZ Zhang, a collaborative study of the epidemiology of rheumatic diseases in China was began in 1984 [1,2]. Subsequently, the Community Oriented Program for Control of Rheumatic Diseases (COPCORD) program was proposed by the World Health Organization (WHO), ILAR and the Asia Pacific League of Associations for Rheumatology (APLAR) [3], and many other surveys were conducted either in cooperation with developed countries such as the USA [4] or by Chinese experts alone. This report reviews the epidemiology of rheumatic disease in China.

## Materials and methods

Reports of population studies relating to the epidemiology of rheumatic diseases from 1980 to 2006 were screened manually and by electronic searches of the Chinese National Knowledge Infrastructure (1980 to 2006), English Medical Current Content (1994 to 2006), Medline (1980 to 2006) and Pub Med (1980 to 2006). The databases was searched using the following search terms: rheumatic disease, rheumatism, rheumatic complaints, osteoarthritis, spondyloarthropathy, ankylosing spondylitis, psoriatic arthritis, reactive arthritis, Reiter syndrome, inflammatory bowel disease arthritis, rheumatoid arthritis, hyperuricemia, gout, systemic lupus erythematosus, primary Sjögren's syndrome, soft tissue rheumatism, fibromyalgia, and epidemiology, China. All of the abstracts were reviewed and relevant reports identified. The findings are presented in seven sections: rheumatic complaints; osteoarthritis (OA); ankylosing spondylitis (AS) and other forms of spondyloarthropathy (SpA); rheumatoid arthritis (RA); hyperuricemia; gout; and other rheumatic diseases. We included only population-based surveys that used the ILAR-China or COPCORD protocols, or other methods (including medical interview, physical examination and laboratory/radiographic examination) employing generally accepted diagnostic criteria, and were published in recognized journals. These reports were extracted and pooled for analysis.

### Diagnostic criteria

The diagnosis of RA and systemic lupus erythematosus (SLE) was made according to the American College of Rheumatology (ACR) criteria available at the time of the study. AS was diagnosed using the New York criteria or the Modified New York criteria, and SpA was identified using the European Spondyloarthropathy Study Group criteria or the Amor criteria. OA was diagnosed on the basis of symptoms plus radiographic features, or physical findings and radiographs, or ACR classification criteria (for hand, knee, and hip OA). Hyperuricaemia was defined as serum uric acid above 7.0 mg/dl in men and above 6.0 mg/dl in women. Gout was diagnosed using the 1977 ACR criteria.

## Results

More than 500 articles were identified, of which 38 surveys [3-41], involving 241,169 individuals from rural or urban areas, fulfilled the requirements for inclusion in this study (Table 1).

**Table 1:** List of 38 reports on common rheumatic diseases in China between 1974 and 2006

Site of survey [ref.]	Location	Age (Years)	Time	Method	Number			Focus
						
					Male	Female	Total	
Taiwan [4]	Rural island	≥17	1974	Other	2,728	2,901	5,629	RA and AS
4 cities [5]	Urban	≥20	1980	Other	267	235	502	SUA
Changchun [6]	Factory	≥20^a,b^	1980	Other	27,272	8,825	36,097	RA and AS
Shanghai [7]	Textile factory	≥18^c^	1984	Other	12,374	20,294	32,668	SLE
Shantou [8]	Rural/urban	≥16	1985	Other	5,632	5,015	10,647	AS
Beijing [9]	Rural	≥20	1987	ILAR	2,090	2,102	4,192	Common RD
Shantou [9]	Rural	≥20	1987	COPCORD	2,384	2,673	5,057	Common RD
Hebei [10]	Coal mine	≥16^b^	1988	Other	892	108	1,000	OA
Beijing [11]	Urban	40 to 58	1988	Other	1,062	951	2,013	SUA
Beijing [11]	Rural	40 to 58	1988	Other	558	949	1,507	SUA
Heilongjiang [12]	Mountain	≥18^a^	1989	Other	1,224	1,087	2,311	Common RD
Guangzhou [13]	Urban/rural	21 to 40	1989	Other	12,102	13,590	25,692	SLE
Ningxia [14]	Highland	≥18^d^	1990	ILAR	5,143	5,277	10,420	Common RD
Shanghai [3]	Urban	≥16	1992	COPCORD	914	1,096	2,010	Common RD
Shantou [15–17]	Urban	≥16	1992	COPCORD	910	812	1,722	OA, BMD and gout
Hong Kong [18]	Urban	≥16	1992	Other	898	1,090	1,988	RA
Taiwan [19]	Urban	≥20	1992	Other	1,534	1,466	3,000	Common RD
Taiwan [19]	Suburban	≥20	1992	Other	1,477	1,523	3,000	Common RD
Taiwan [19]	Rural	≥20	1992	Other	1,555	1,443	2,998	Common RD
Beijing [20]	Rural	≥16^c^	1994	Other	653	1,410	2,063	Knee OA
Beijing [21]	Suburban	≥16^c^	1994	Other	611	1,359	2,066	pSS
Taiwan [22]	Mountain	≥18^e^	1994	Other	145	197	342	SUA and gout
Shantou [23]	Urban	≥16	1995	COPCORD	985	1,055	2,040	Common RD
Taiwan [24]	Island	≥19	1996	Other	2,754	2,953	5,707	SUA and gout
Shandong [25]	Rural	≥16	1996	ILAR	2,695	2,360	5,055	RA and AS
Shanghai [26]	Urban	≥15	1997	COPCORD	913	1,124	2,037	SUA and gout
Shandong [27]	Coast	≥20	1997	Other	8,449	8,595	17,044	Common RD
Shanghai [28]	Urban	≥16	1998	COPCORD	3,190	3,394	6,584	Common RD
Shantou [29,30]	Suburban	≥16	1999	COPCORD	975	1,054	2,029	SpA and gout
Beijing [31]	Urban	≥16	1999	COPCORD	1,025	957	1,982	SpA
Beijing [32]	Urban	≥60	2000	Other	614	878	1,492	Hip OA
Beijing [33]	Urban	≥60	2000	Other	1,004	1,503	2,507	Hand OA
Beijing [34]	Urban	≥60	2000	Other	730	1,051	1,781	Knee OA
Northeast [35]	15 Provinces^f^	17 to 40^g^	2000	COPCORD	20,068	0	20,068	AS
Qingdao [36]	Urban	≥20	2002	Other	720	1,303	2,023	SUA
Nanjing [37]	Urban	≥20	2003	Other	3,849	4,039	7,888	SUA and gout
Shanghai [38]	Urban	≥40	2003	COPCORD	894	1,199	2,093	Knee OA
Taiyuan [39–41]	Urban	≥16	2004	COPCORD	1,858	2,057	3,915	Common RD

All mentions of 'OA' in this table refer to symptomatic osteoarthritis (OA). ^a^Han and Manchu ethnic. ^b^Male>Female. ^c^Male<Female. ^d^Han and Muslim ethnic. ^e^Aborigines. ^f^11 ethnic. ^g^All male. AS, ankylosing spondylitis; BMD, bone mineral density; COPCORD, Community Oriented Program for Control of Rheumatic Diseases; F, female; ILAR, International League of Associations for Rheumatology; M, male; pSS, primary Sjögren's syndrome; RA, rheumatoid arthritis; RD, rheumatic diseases; SLE, systemic lupus erythematosus; SUA, serum uric acid; SpA, spondyloarthropathy.

### Distribution of study locations

**Figure 1:**
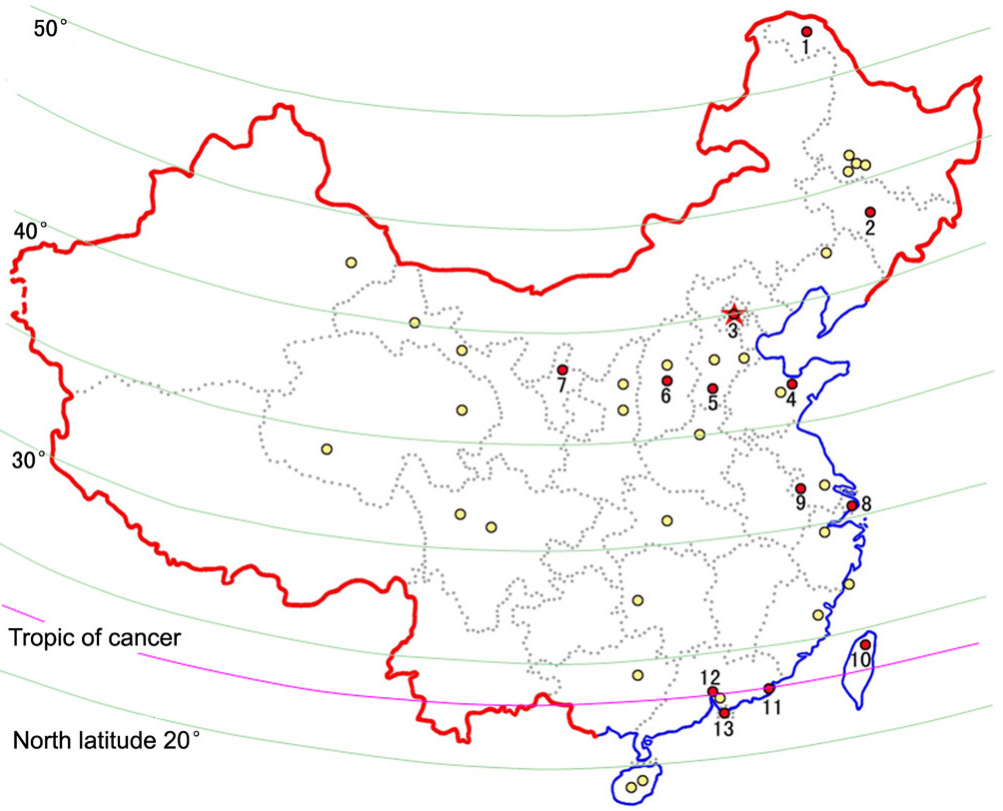
Targeted areas of the epidemiological study in China. The red circles indicate the locations of surveys of common rheumatic diseases. The yellow circles indicate the locations of hand OA surveys. 1, Heilongjiang; 2, Jilin; 3, Beijing; 4, Shandong; 5, Hebei; 6, Taiyuan; 7, Ningxia; 8, Shanghai; 9, Nanjing; 10, Taiwan; 11, Shantou; 12, Guangzhou; 13, Hong Kong.

Thirty-eight surveys were analyzed. These were conducted in 25 provinces/cities, covering an area from northeast (Heilongjiang) to southeast (Hong Kong), and from northwest (Ningxia) to east (Taiwan) of China (Figure 1).

### Rheumatic complaints

The prevalence of rheumatic complaints, as reported in 13 surveys [3,9,12,15,18,19,23,28,30,41] that included 40,635 adult in seven provinces/cities, varied from 11.6% to 46.4% (Table 2). These complaints were more prevalent in women than in men, were more frequently observed in elderly than in young individuals, and were more common in the north than in the south. In the Shantou area there has been an increase in prevalence of rheumatic complaints during the past decade. The rise in prevalence with latitude previously described [42] receives support from the study findings evaluated here.

**Table 2:** Rheumatic complaints in general populations

Site of survey [ref.]	Location	Age (Years)	Latitude	Time	Number			Prevalence (%)		
					
					Male	Female	Total	Male	Female	Total
Heilongjiang [12]	Rural	≥18	46	1989	1,224	1,087	2,311	47	46	46.4
Beijing [9]	Rural	≥20	40	1987	2,090	2,102	4,192	33.4	47.1	40.3
Taiyuan [41]	Urban	≥16	38	2004	1,858	2,057	3,915	15.23	20.89	18.4
Shanghai [3]	Urban	≥16	32	1992	914	1,096	2,010	16.9	30.5	24.3
Shanghai [28]	Urban	≥16	32	1998	3,190	3,394	6,584	11	18.7	13.3
Taiwan [19]	Urban	≥20	25	1992	1,534	1,466	3,000	22	31	26.3
Taiwan [19]	Suburban	≥20	25	1992	1,477	1,523	3,000	14.5	22.5	18.4
Taiwan [19]	Rural	≥20	25	1992	1,555	1,443	2,998	17	32	24.3
Shantou [9]	Rural	≥20	23	1987	2,384	2,673	5,057	8.9	14	11.6
Hong Kong [18]	Urban	≥16	22	1992	898	1,090	1,988	9.5	15.8	13.0
Shantou [15]	Urban	≥16	23	1992	910	812	1,722	11.8	13.3	12.5
Shantou [23]	Urban	≥16	23	1995	985	1,055	2,040	12.3	21	18.1
Shantou [30]	Suburban	≥16	23	1999	863	955	1,818	15.9	23.1	19.8

### Symptomatic osteoarthritis

Thirteen surveys involving 29,621 adult people from six regions reported on the prevalence of symptomatic OA [3,10,16,19,20,23,32-34,38,39], which ranged from 5.1% to 20.8% (Table 3). The lowest rate was in a urban area of Taiwan [19], and the highest rate was reported in a survey of coal miners in Handan of Hebei province [10].

**Table 3:** Prevalence of symptomatic osteoarthritis in China

Site of survey [ref.]	Location	Age (Years)	Time	Number	Prevalence (%)									
					
					Total	Knee	Lumbar	Cervical	Shoulder	Elbow	Hand	Feet	Ankle	Hip
Hebei [10]	Coal mine	≥16^a^	1988	1,000	20.8	1.5	12.9	1.4		1.3	0.50	0.30	0.10	2.0
Taiyuan [39]	Urban	≥16	2004	3,915	11.15	7.57	3.60	3.54			0.80			0.03
Shantou [16]	Urban	≥16	1992	1,722	8.3	1.3	6.0	4.5	0.06	0	0.1	0.3		0.15
Shantou [23]	Urban	≥16	1995	2,040	10.8	3.2	7.6	1.8	0.2	0.4	0.4	0.1	0.3	0.15
Shanghai [3]	Urban	≥16	1992	2,010	13.0									
Taiwan [19]	Urban	≥20	1992	3,000	5.1									
Taiwan [19]	Suburban	≥20	1992	3,000	5.8									
Taiwan [19]	Rural	≥20	1992	2,998	6.3									
Beijing [20]	Rural	≥16	1994	2,063		9.6								
Beijing [32]	Urban	≥60	2000	1,492										0.07
Beijing [34]	Urban	≥60	2001	1,781		11.1								
Shanghai [38]	Urban	≥40	2003	2,093		7.2								
Beijing [33]	Urban	≥60	2001	2,507							M 3.0/F 5.8			

^a^Male (M) > Female (F).

The most common sites of OA in these reports were lumbar spine, knee joints and cervical spine. The lowest prevalence of lumbar OA (3.6%) was identified in an urban area of Taiyuan [39], and the highest rate (12.9%) in the population of coal miners [10]. Regarding the prevalence of knee OA, the lowest rate (1.3%) was identified in a Shantou school population [16], and the highest (11.1%) in a Beijing elderly urban population [34]. The lowest prevalence of cervical OA (1.4%) was identified in the coal miners [10], and the highest (4.5%) was reported from the Shantou school population [16]. Apart from these common sites of OA involvement, the prevalence rates of hip OA and elbow OA in the coal miner population were 2.0% and 1.3% [10]; the rates of hand OA among Beijing residents aged 60 years or older were 3.0% and 5.8% in men and women, respectively [33]. OA in other sites such as shoulder, elbow, hand, feet, ankle and hip were rarely observed.

### Ankylosing spondylitis and the other forms of spondyloarthropathy

Seventeen surveys, including 120,451 adults from 12 provinces/cities, reported on the prevalence of AS [3,4,6,8,9,12,14,19,23,25,30,31,35,40] (Table 4). Fourteen of these 17 surveys were conducted in Han ethnic populations, and the prevalence was 0.2% to 0.54%. The other three surveys were conducted in mixed ethnic populations in Ningxia (mainly of Muslim and Han) [14], Heilongjiang (Manchu and Han) [12], and Changchun (Manchu and Han) [6], and the prevalence rates were only 0.10%, 0.09% and 0.06%, respectively. Nevertheless, a rate of 0.24% was reported from a survey conducted in 20,068 male soldiers aged 17 to 40 years and selected from 11 ethnic origins [35].

**Table 4:** Prevalence of spondyloarthropathy in China

Site of survey [ref.]	Location	Age (Years)	Ethnic	Time	Number	Prevalence (%)			
						
						AS	PsA	ReA	uSpA
Taiwan [4]	Island	≥17	Han	1974	5,629	0.2			
Beijing [9]	Rural	≥20	Han	1987	4,192	0.26			
Beijing [31]	Urban	≥16	Han	1999	1,982	0.3	0.10		1.21
Taiyuan [40]	Urban	≥16	Han	2004	3,915	0.2	0.05		0.64
Shandong [25]	Rural	≥16	Han	1996	5,055	0.22	0.04	0.02	
Shanghai [3]	Urban	≥16	Han	1992	2,010	0.20			
Taiwan [19]	Urban	≥20	Han	1992	3,000	0.4			
Taiwan [19]	Suburban	≥20	Han	1992	3,000	0.19			
Taiwan [19]	Rural	≥20	Han	1992	2,998	0.54			
Shantou [8]	Rural	≥16	Han	1985	10,647	0.20			
Shantou [9]	Rural	≥20	Han	1987	5,058	0.26			
Shantou [23]	Urban	≥16	Han	1995	2,040	0.2			
Shantou [30]	Suburban	≥16	Han	1999	2,029	0.3	0.05		1.0
Military [35]	15 provinces	17 to 40^a^	11 ethnics	2000	20,068	0.24			
Heilongjiang [12]	Mountain	≥18	H and Ma^c^	1989	2,311	0.09			
Changchun [6]	Factory	≥20^b^	H and Ma^c^	1980	36,097	0.06	0.01		
Ningxia [14]	Highland	≥18	H and Mu^d^	1990	10,420	0.10			

^a^All male. ^b^Male > female. ^c^Han and Manchu. ^d^Han and Muslin. AS, ankylosing spondylitis; PsA, psoriatic arthritis; ReA, reactive arthritis; uSpA, undifferentiated spondyloarthropathy.

The prevalence of PsA was 0.01% to 0.1% [6,25,30,31,40], reactive arthritis 0.02% [25], and undifferentiated spondyloarthropathy (uSpA) 0.64% to 1.2% [30,31,40]. No case of inflammatory bowel disease arthritis was reported.

#### Frequency of HLA-B27

The frequency of *HLA-B27 *positive status in the Han ethnic general population ranged from 3.6% to 5.7% [8,43-46]. The frequency of *HLA-B27 *positive status in patients with AS ranged from 90.6% to 93.6% [8,47,48]. At least eight subtypes of *HLA-B27 *have been identified. The most common subtypes were *B2704 *and *B2705 *[49-52]. No data on minority ethnic populations have been reported.

Family surveys of *HLA-B27 *positive AS revealed that about half of first-degree relatives were *HLA-B27 *positive, among whom the likelihood of developing AS was 50% [8,53,54].

### Rheumatoid arthritis

Fifteen surveys from 10 provinces/cities, involving 94,297 adults, reported on the prevalence of RA (Table 5) [3,4,6,9,12,14,18,19,23,25,28,41]. In mainland China the prevalence ranged from 0.2% to 0.37%, and no significant difference was noted between north and south or between different ethnic groups. In the Taiwan Island [19], a higher prevalence of RA of 0.93% was reported from an urban area, but in a rural area it was 0.26%, similar to that reported from the mainland.

**Table 5:** Prevalence of rheumatoid arthritis in China

Site of survey [ref.]	Location	Age (Years)	Ethnic	Time	Number	Prevalence (%)
Taiwan [4]	Rural island	≥17	Han	1974	5,629	0.3
Beijing [9]	Rural	≥20	Han	1987	4,192	0.34
Taiyuan [41]	Urban	≥16	Han	2004	3,915	0.28
Shandong [25]	Rural	≥16	Han	1996	5,055	0.36
Shanghai [3]	Urban	≥16	Han	1992	2,010	0.2
Shanghai [28]	Urban	≥16	Han	1998	6,584	0.28
Hong Kong [18]	Urban	≥16	Han	1992	1,988	0.35
Shantou [9]	Rural	≥20	Han	1987	5,058	0.32
Shantou [23]	Urban	≥16	Han	1995	2,040	0.2
Taiwan [19]	Urban	≥20	Han	1994	3,000	0.93
Taiwan [19]	Suburban	≥20	Han	1994	3,000	0.78
Taiwan [19]	Rural	≥20	Han	1994	2,998	0.26
Heilongjiang [12]	Mountain	≥18	H and Ma^a^	1989	2,311	0.50
Changchun [6]	Factory	≥20	H and Ma^a^	1980	36,097	0.32
Ningxia [14]	Highland	≥18	H and Mu^b^	1990	10,420	0.36

^a^Han and Manchu. ^b^Han and Muslin.

### Hyperuricaemia and gout

During the early 1980s, the prevalence of hyperuricaemia (Table 6) was only 1.4% in males and 1.3% in females in Beijing, Shanghai, Hangzhou and Guangzhou [5]. In 1987 to 1988, however, the corresponding figures rose to 15.4% and 11.3% in Beijing [11] and 14.2% and 7.1% in Shanghai. In mainland China, the highest prevalence was reported from Qingdao [36], with rates of 32.1% in men and 21.8% in women. Nevertheless, these rates were much lower than those reported in Taiwan aborigines, at 53.8% in men and 30.7% in women [22].

**Table 6:** Prevalence of hyperuricemia and gout in China

Site of survey [ref.]	Location	Age (Years)	Time	Number	Hyperuricaemia^a ^(%)	Gout (%)
Four cities [5]	Urban	≥20	1980	502	M 1.4/F 1.3	0
Beijing [11]	Urban	40 to 58	1988	2,013	M 15.4/F 11.3	
Beijing [11]	Rural	40 to 58	1988	1,507	M 11.0/F 8.4	
Qingdao [36]	Urban	≥20	2002	2,023	M 32.1/F 21.8	0.36
Nanjing [37]	Urban	≥20	2003	7,888	M 13.8/F 6.1	0.67
Shanghai [26]	Urban	≥15	1997	2,037	M 14.2/F 7.1	0.34
Taiwan [24]	Island	≥19	1996	5,707	M 42.1/F 27.4	1.98
Taiwan [22]	mountain	≥18^b^	1994	342	M 53.8/F 30.7	11.7
Taiyuan [41]	Urban	≥16	2004	3,915		0.15
Shanghai [3]	Urban	≥16	1992	2,010		0.2
Shanghai [28]	Urban	≥15	1998	6,584		0.22
Shantou [17]	Urban	≥16	1992	1,722		0.17
Shantou [23]	Urban	≥16	1995	2,040		0.15
Shantou [29]	Urban	≥16	1999	1,818		0.26
Taiwan [19]	Urban	≥20	1992	3,000		0.67
Taiwan [19]	Suburban	≥20	1992	3,000		0.67
Taiwan [19]	Rural	≥20	1992	2,998		0.16

^a^Male (M) ≥ 7.0 mg/dl, female (F) ≥ 6.0 mg/dl. ^b^Aborigines.

Since 1992 the prevalence of primary gout has been reported in 14 surveys [3,17,19,22-24,26,28,29,36,37,41] in 11 areas involving 45,084 adults (Table 6). In mainland China, it ranged from 0.15% to 0.67% in Han Chinese. The prevalence was high in Taiwan aborigines [22], at 11.7%. In contrast, the prevalence of gout in a Taiwan rural area Han ethnic population survey [19] was only 0.16%, similar to that reported in mainland China.

The prevalence rates of primary gout in the Shantou area in 1992, 1995 and 1999 were 0.17%, 0.15% and 0.26%, respectively [29], which indicates a trend toward increased prevalence in the 1990s. Yang and coworkers [55] analyzed changes in incidence of primary gout in 21 hospitals situated throughout northern to southern China during the period from 1979 to 1993; in all cases they found a trend toward increased incidence, which was more evident in southern cities. This was in accordance with findings in Shanghai; in the latter, although no case of gout was found in the survey conducted in the 1980s, the prevalence has been more than 0.2% since 1992.

### Systemic lupus erythematosus

Three surveys were carried out in 1984, 1989 and 1997 in Shanghai [7], Guangzhou [13] and Shandong [27]. The surveyed population sizes were 32,668, 25,692 and 17,044, and the obtained prevalence rates of SLE were 0.07% (70.1/100,000), 0.03% (31.1/100,000) and 0.05% (46.5/100,000), respectively.

### Soft tissue rheumatism

Soft tissue rheumatism was surveyed in Shantou, Guangdong province, and Taiyuan, Shanxi province in 2005 [41]. The prevalence in Shantou was 5.7%, which is significantly higher than that in Taiyuan (2.5%). Rotator cuff tendinitis, adhesive capsulitis (frozen shoulder) and lateral epicondylitis (tennis elbow) were the most frequent soft tissue rheumatism diagnoses, with prevalence rates of 0.8%, 0.5% and 0.6%, respectively. The prevalence was significantly higher in women than in men (4.7% versus 2.6%) and exhibited an increasing trend with age, particularly in the 35 to 54 years age group. Fibromyalgia was seldom seen in both these areas; only two cases were found in Shantou (2,350 people) and one in Taiyuan (3,915 people).

### Primary Sjögren's syndrome

In 1995 Zhang and coworkers [21] reported a population survey of 2,066 adults in Beijing, which indicated the prevalence of primary Sjögren's syndrome to be 0.77% by the Copenhagen classification criteria and 0.33% by the San Diego classification criteria.

## Discussion

During the past two decades many studies of the epidemiology of rheumatic diseases in China have been conducted. The authors fully appreciate the difficulties in summarizing all of these reports, because most of them were not done in a uniform or systematic way. Because of these issues, only 38 surveys – including 241,169 adults from 25 provinces/cities – were pooled and analyzed. Evidently, the surveyed populations were living under different environment conditions, and age and sex distributions were diverse. Potential bias, resulting for instance from methodological problems, differences in age of the people surveyed, interobserver error, and so on, would certainly have influence the survey results. However, the key procedure in estimating disease prevalence was similar for all of these surveys, which included medical interview, physical examination and relating laboratory/radiographic examinations. Furthermore, the diagnostic criteria for the diseases considered were all generally acceptable. The major difference between studies employing the COPCORD protocol and the other studies was that the former included additional information related to the burden of disease, intervention and aetiology of disease. Most surveys included here aimed to assess prevalence rates of different rheumatic diseases, with a few including an evaluation of risk factors.

Prevalence rates of rheumatic pain reported from Australia, Bangladesh, India, Indonesia, Philippines, Thailand and Vietnam were 33%, 26.3%, 18.2%, 23.6% to 31.3%, 16.3%, 36.2% and 14.9%, respectively [56-62], indicating variation by locality, methods of survey, definition of disease categories and ethnic group. As shown in Table 2, there was a trend for the rate of positive response in the general population to a questionnaire relating to rheumatic complaints to increase with increasing latitude in locality. In Malaysian Chinese living at 5° north latitude, the prevalence of rheumatic complaints was only 13% in a 1992 survey [63]. Furthermore, in the Shantou area it was shown that after the 1980s, following growing economic development, there was a trend toward increased prevalence of rheumatic pain [42]. It is thus of importance to investigate how socioeconomic status, environmental differences, sex, age, occupation, ergonomics, bone mineral density and awareness of seeking medical care influence the prevalence of rheumatic complaints.

The prevalence of symptomatic OA varied widely with locality and population. In China the prevalence of OA ranged from 5.1% to 20.8%, with a mean of 9.1%. This was close to that reported in the other Asia Pacific countries such as Australia [56], Thailand [61], Vietnam [62], India [64] and Philippines [65] (5.5%, 11.3%, 4.1%, 5.8% and 4.1%, respectively). It is notable that in coal miners the prevalence was as high as 20.8% [10] (Table 3). Although the prevalence of hip OA was very low in Chinese as compared with UK and US Caucasians [32], the prevalence of hip OA was higher among the Chinese coal miners [10] (2.0%) than in the general population, as was found in UK miners by Lawrence [66]. This is apparently related to the heavy physical labour of the miners. In elderly Chinese males the prevalence of knee OA was comparable to that in Caucasians, but for elderly Chinese women the prevalence of knee OA was higher than in Caucasian women [34,67]. In 1995 Zeng and colleagues [68] reported that, in both clinical and epidemiological studies, the prevalence of symptomatic hand OA in Shantou, China (0.4%) was markedly lower in Chinese than in Caucasians. Reports from Hebei in 1988 [10] and Taiyuan in 2004 [39], and a cooperative Chinese-American study of hand OA (the ratio of hand OA prevalence in China to that in the USA was 0.25%, after adjustment for age) [33], further enhanced the impression that the prevalence of hand OA was indeed lower in Chinese than in Caucasians.

In China, the prevalence of AS among the Han ethnic population (0.2% to 0.54%) was close to that in Caucasians [69,70] but higher than that in Thailand (0.12%) [61]. However, among mixed ethnic populations such as Manchu and Han or Muslin and Han, the prevalence of AS (about 0.1%) was lower than that in the Han ethnic group. Although *HLA-B27 *was not investigated in these surveys, these findings once again suggest that genetic factors are associated with AS. Further study including *HLA-B27 *tests in individual minority ethnic populations is necessary to confirm these findings. uSpA was a frequently neglected form of spondylopathy. The prevalence of uSpA ranged from 0.64% to 1.21% in the Han ethnic group, even higher than that of frank AS, which challenges clinicians to improve their diagnostic awareness of uSpA.

The prevalence of RA in mainland China, ranging from 0.2% to 0.37%, was similar to that in most Asian countries [60,61,64,65,71] and South American countries [72,73] but lower than that in Caucasians [74]. In Taiwan urban and suburban areas, the prevalence of RA (0.93%) was closer to rates in Caucasians, but the prevalence of 0.26% in a Taiwan rural area was similar to that in mainland China. This might be accounted for by the fact that Taiwan urban areas were more developed than in mainland China. Apart from genetic factors, environmental and socioeconomic factors might be important risk factors for RA; this possibility awaits further study.

For some time, hyperuricaemia and gout were thought to be rare in China [5]. Since the 1980s it has become apparent that the prevalence of hyperuricaemia has exhibited a trend toward increased prevalence in both men (from 11.0% to 32.1%) and women (from 6.1% to 21.8%). Among Taiwan aborigines, the high rates of hyperuricaemia of 53.8% in men and 30.7% in women are remarkable. In mainland China, the prevalence of gout (0.15% to 0.67%) is lower than in Austronesians (Malayo-Polynesians) [75,76], Caucasians [77] and Australians [56], and slightly higher than in some other Asia countries such as Thailand, Vietnam and India (0.16%, 0.14% and 0.12%, respectively) [61,62,64]. Data from the USA showed that the overall prevalence of gout had doubled from 1969 to 1986 [78]. This trend toward a general increase indicates that living in affluent populations, and consequent changes in dietary habits and lifestyle are the main risk factors for gout. Improvement in diagnostic measures for gout may also play a role.

Prevalence of soft tissue rheumatism (2.5% to 5.7%) in China was close to that in some Asian countries such as Bangladesh (2.5%) [57] and Philippines (3.8%) [60]. Fibromyalgia was rarely observed in China, with a prevalence of 0.05 % (3/6,265), which was distinctly lower than in reports from other parts of the world [57,79]. Veerapen reported from Malaysia in 1992 [63] that the prevalence of fibromyalgia was higher among Indian than among Malay, and lowest among Chinese. This was in accordance with the situation in mainland China. Buskila and coworkers [80] claimed that fibromyalgia was associated with genetic factors. Whether the low prevalence of fibromyalgia in China has any genetic explanation awaits further study.

Many population surveys of the prevalence of SLE have been conducted in China [81]. The minimum sample size required is considered to be 30,000. In the Guangzhou and Shandong surveys the prevalence rates were 0.031% (8/25,692) and 0.053% (9/17,044), yielding a combined prevalence of 0.036% (17/46,736), which is similar to US Caucasian prevalence rates, which range from 0.0146% to 0.124% [82]. The Shanghai survey of textile factory workers revealed a higher prevalence at 0.07% (23/32,626). There was a higher proportion of women to men in the Shanghai survey (1.6:1), and so the population prevalence would have been overestimated. The factory workers might have been exposed to risk factors that are specific to that environment, such as chemical exposure.

The only survey of primary Sjögren's syndrome suggested that it was not rare in China, but many cases had previously been overlooked or misdiagnosed.

## Conclusion

Rheumatic diseases are common in China. Prevalence of rheumatic symptoms increases with latitude and varies with the locality and age. The prevalence of OA was comparable with that in Western countries but exhibited variance in joint distribution; the highest rates for hip and knee OA were in coal miners. The prevalence of AS in China was similar to that in Caucasians and similarly related to HLA type. The prevalence of RA was lower than that in the developed countries except in a more developed Taiwan urban area. The prevalence of hyperuricaemia has increased since 1990. Although the prevalence of gout was lower than in the developed countries, there has been a trend toward increased prevalence in China in recent years. Fibromyalgia was rarely seen in China. China includes a vast territory with more than 50 ethnic groups. Most minority groups live in the west and northwest parts of China. Paying more attention to the epidemiology of rheumatic diseases in these areas is of great importance.

## Abbreviations

APLAR = Asia Pacific League of Associations for Rheumatology; AS = ankylosing spondylitis; COPCORD = Community Oriented Program for Control of Rheumatic Diseases; ILAR = International League of Associations for Rheumatology; OA = osteoarthritis; RA = rheumatoid arthritis; SLE = systemic lupus erythematosus; SpA = spondyloarthropathy; uSpA = undifferentiated spondyloarthropathy; WHO = World Health Organization.

## Competing interests

The authors declare that they have no competing interests.

## Authors' contributions

QYZ participated in the design and prepared the manuscript, and took part in the ILAR-China study and WHO ILAR-APLAR COPCORD Shantou, Beijing, Taiyuan study. RC and ZYX participated in the design, and took part in the ILAR-China study and WHO ILAR-APLAR COPCORD Shantou, Beijing, Taiyuan study. SBC took part in the data collection, performed the statistical analyses and helped to prepare the manuscript. JD and RW participated in the design, helped to finalize the manuscript, were the supervisors of the WHO ILAR-APLAR COPCORD study, and took part in the ILAR-China study and WHO ILAR-APLAR COPCORD Shantou, Beijing study. SLC and NZZ directed the ILAR-China study and WHO ILAR-APLAR COPCORD study, respectively, and helped to prepare the manuscript. All authors read and approved the final manuscript.
